# Recent Advances in Enteral Nutrition

**DOI:** 10.3390/nu8110709

**Published:** 2016-11-08

**Authors:** Omorogieva Ojo, Joanne Brooke

**Affiliations:** 1 Senior Lecturer in Primary Care, Faculty of Education and Health, University of Greenwich, London SE9 2UG, UK; 2 Reader in Complex Older Persons care Oxford Institute of Nursing and Allied Health Research, Faculty of health and Life Sciences, Oxford Brookes University, Oxford OX3 0FL, UK; jbrooke@brookes.ac.uk

There have been significant advances in the provision of enteral nutrition support in the acute and community healthcare settings. Enteral nutrition is beneficial to individuals who have functional guts but may not be able to meet their nutritional requirements via a normal diet. Most of these people have neurological conditions such as stroke, multiple sclerosis and dementia which could impact on swallowing reflexes, leading to dysphagia [1]. Others may have cancer, intellectual disability or conditions such as HIV and failure to thrive.

Therefore, the provision of nutrition support in the form of oral nutritional supplements (ONS) and enteral nutrition support can help mitigate the challenges of nutritional deficit [2]. Enteral feeding can be delivered via a range of feeding tubes and through different methods of feeding including continuous, bolus and gravity feeding [3]. While nasogastric tube (NGT) feeding is often provided to individuals requiring short-term enteral nutrition provision, the percutaneous endoscopic gastrostomy (PEG) tube is for long-term enteral feeding [4]. For individuals with partial/complete gastrectomy and those who are at higher risk of aspiration, the use of the jejunostomy feeding tube may help alleviate these problems [5]. On the other hand, the radiologically inserted gastrostomy (RIG) tube may be the tube of choice in head and neck cancer patients who may have high risk of malignant cell translocation from the primary site of disease to the stoma site. Similarly, the use of the balloon gastrostomy feeding tube, following the dislodgement of the conventional enteral feeding tube (PEG, RIG), is common, although there is evidence that the balloon gastrostomy feeding tube is now used as a primary tube of choice in head and neck cancer patients [6].

Usually, the provision of enteral nutrition entails nutritional status assessment and the evaluation of nutritional requirements of patients [7]. In addition, the development of feeding regimes, protocols, guidelines, algorithms, and the management of patients, pumps, feeds, and feeding tubes are essential aspects of enteral nutrition provision.

The developments in enteral nutrition appear to center on many aspects, including the increasing use of enteral feeding in patients with long-term conditions, the development of multidisciplinary teams including extended roles for dietitians and nurses and the use of guidelines. This may not be unrelated to the worldwide increase in the aging population and increasing prevalence of long-term conditions with associated complications, resulting in swallowing difficulties and malnutrition [8].

Therefore, the essence of the Special Issue on Recent Advances in Enteral Nutrition was to capture key developments in this area of research and practice. For instance, dementia is a long-term condition that impacts on people’s cognitive and physical abilities which can affect their nutritional intake, leading to malnutrition [9]. Malnutrition in patients with dementia appears to correlate with cognitive decline and the progression of the disease. The use of percutaneous endoscopic gastrostomy which is used widely in supporting patients with a range of conditions seems to be discouraged in dementia care [10]. In a systematic review on the use of enteral nutrition in patients with advanced dementia, Finucance et al. [10] did not find any improvements in the rates of aspiration, pressure sores and mortality and therefore concluded that enteral nutrition for patients with dementia should be discouraged [10]. In contrast, recent recommendations from the systematic review by Brooke and Ojo [9] challenged this position and instead suggested the need for a holistic assessment of patients with dementia requiring enteral nutrition and PEG tube placement. These assessments should include a diagnosis of patients—comorbidities, current stage of dementia, acute medical illness and its impact on nutritional status [9].

Another area where enteral nutrition is being used to support patients with a long-term condition is in diabetes care and management. The complications of diabetes are wide ranging and may include stroke, which could impact on the swallowing ability of the individuals [4]. The use of enteral nutrition to support these people who are unable to meet their nutritional requirements via oral intake alone becomes imperative [11]. Therefore, Ojo and Brooke [12] evaluated the use of standard and diabetes specific enteral formulas in the management of diabetes in a systematic review. Based on the response of blood glucose and other parameters including HBA1c in the studies reviewed, it was concluded that the use of diabetes specific formula may be effective in managing glucose in patients with diabetes and on enteral nutrition [12]. 

There have been advances in the use of enteral nutrition to support patients with head and neck cancer and other cancers through the use of different feeding tubes, both as prophylactic and reactive treatments [13,14]. Patients with head and neck cancer are mostly malnourished and/or at risk of malnutrition, therefore, prophylactic feeding through NGT or PEG aimed at improving weight gain and promoting hydration is now common [15]. However, based on the narrative review by Bossola [15], it would appear that the use of prophylactic enteral feeding does not offer advantages with respect to nutritional outcomes, effect on radiotherapy treatment and survival compared with reactive feeding, which involves patients being offered NGT or PEG when oral nutritional supplements are inadequate in maintaining nutritional status [15]. 

In another study, Wang et al. [16] compared postoperative enteral nutrition with delayed enteral nutrition in patients with oesophageal cancer with a view to establishing the most appropriate time to commence enteral nutrition provision. It was concluded that early enteral nutrition started within 48 hours was safe for postoperative oesophageal cancer patients [16]. Based on this study, it was shown that early enteral nutrition is effective in reducing the incidence of postoperative pulmonary infection, promoting postoperative nutrition status, enhancing early recovery of intestinal movement and reducing the length of hospital stay and hospital cost [16]. 

Apart from patients with head and neck cancer, enteral nutrition is also used to support patients with other forms of cancer including pancreatic cancer. According to Buscemi et al. [17], pancreaticoduodenectomy is used for the treatment of periampullory carcinomas and patients who have undertaken this procedure are often malnourished with significant impact on postoperative wound healing and recovery. Following this review, it was concluded that enteral nutrition appeared safe and tolerated by patients who have had pancreaticoduodenectomy although it did not provide any advantage in terms of postoperative pancreatic fistula, postpancreatectomy haemorrhage, length of hospital stay and infectious complications [17]. 

Inflammatory bowel disease, which includes at least three clinical conditions (ulcerative colitis, Crohn’s disease and indeterminate colitis), is another condition that may benefit from advances in enteral nutrition support [18]. There is evidence that malnutrition is a common effect of inflammatory bowel disease and diet has been implicated in its pathogenesis and clinical manifestation [18]. In addition, diet also has a role in the management of inflammatory bowel disease and the need for enteral nutrition support becomes critical when oral dietary intake is not sufficient to offer all the nutritional requirements [19]. Enteral nutrition has shown promising results in the management of Crohn’s disease as it provides equal or higher remission rates than current medications in use [18].

In a related study, exclusive enteral nutrition—the monotonous enteral delivery of complete liquid nutrition—has been explored in the management of Crohn’s disease [19]. Exclusive enteral nutrition is usually in the form of liquid enteral formulas which may be elemental (e.g., in the form of amino acids) or polymeric (e.g., in the form of intact protein) [19]. Although the mechanism of action of exclusive enteral nutrition is still evolving, there is evidence that it could modify the composition of intestinal microbiome which are essential in the pathogenesis of Crohn’s disease [19]. It would appear that exclusive enteral nutrition is better than steroids in the induction of mucosal healing and may provide long-term remission in some cases of Crohn’s disease [19].

The efficacy and safety of the use of an enteral immunomodulatory diet (omega-3 fatty acid, γ-linolenic acid and antioxidant supplementation) for acute lung injury and acute respiratory distress syndrome are also areas of interest in enteral nutrition provision. This view relies on the understanding that this therapy may be used for the treatment of these conditions, although researchers are not unanimous on this position [20]. Based on the current systematic review [20], it is now clear that an enteral immunomodulatory diet could not reduce the severity of acute lung injury and acute respiratory distress syndrome.

In Very Low Birth Weight (VLBW) infants, feeding methods in enteral nutrition have been explored based on the observation that continuous enteral feeding methodmay result in significant loss of fat and micronutrients [21]. Therefore, Tabata et al. [21] examined the fat loss in enteral nutrition based on the current methods of providing fortified human milk in high risk infants. In addition, the study evaluated whether fortifier and cream improved fat delivery in continuous enteral infant feeding of breast milk [21]. Based on this study, it was clear that fat and nutrient loss in continuous enteral feeding was presenting a challenge to the provision of nutrients to Very Low Birth Weight infants [21]. Therefore, the bolus feeding method is recommended where possible and for infants who are unable to tolerate bolus feeding, the addition of fortifiers and/or cream to human milk, in order to increase fat percentage, is recommended [21].

The use of human milk fortified with donor human milk-derived fortifier (HMDF) in premature infants has been reported to increase serum phosphorus although the evidence appears anedoctal [22]. Therefore, the study by Chetta et al. [22] investigated this phenomenon and concluded that the incidence of elevated serum phosphorus was mild and not permanent in premature infants receiving human milk with HMDF.

Despite the merits of enteral nutrition, there are a number of challenges militating against the use of enteral feeding. These include problems of funding, inadequate or lack of standards, policies, management approaches, guidelines and infrastructure for the delivery of enteral nutrition [23]. Therefore, strategies for ameliorating these challenges should include the development of the Home Enteral Nutrition (HEN) service which should promote multi-disciplinary team working and the development of national and international standards and guidelines [23]. The National Institute for Health and Care Excellence (NICE) guidance on nutrition support [24] emphasizes the quality standard for nutrition support in adults and stresses the need for all care services to be responsible in identifying those who are at risk of malnutrition and providing nutrition support for the people who need it. In addition, Dutta et al. [25] conducted a comprehensive literature review and developed a set of guidelines for feeding Very Low Birth Weight (VLBW) infants. It was concluded that there is a need to aim for full feeds at about 2 weeks of age in neonates weighing <1000 g at birth and for 1 week in those neonates weighing 1000–1500 g at birth [25]. The use of trophic feeds (10–15 mL/kg/day) should commence within 24 h of birth although caution is required in extremely pre-term, extremely low birth weight and infants with growth restriction [25].

The development of multidisciplinary teams, including primary care teams involved in enteral nutrition provisions, has been shown to improve cost effectiveness [26]. A Home Enteral Nutrition team comprising dietitians, nurses and speech and language therapist has the potential to improve patient satisfaction and reduce the costs which are associated with enteral tube feeding in the community [27]. This is often achieved through the development and implementation of care pathways for the management of patients on enteral tube feeding by the HEN team and effective multidisciplinary team working [26]. The use of the HEN service has increased significantly in the past few decades and this has led to the development of various policies and guidelines for the management of enteral nutrition [28]. This has also contributed to the promotion of multidisciplinary team working and the extension of roles of the different professionals that make up the HEN team [27,29].
